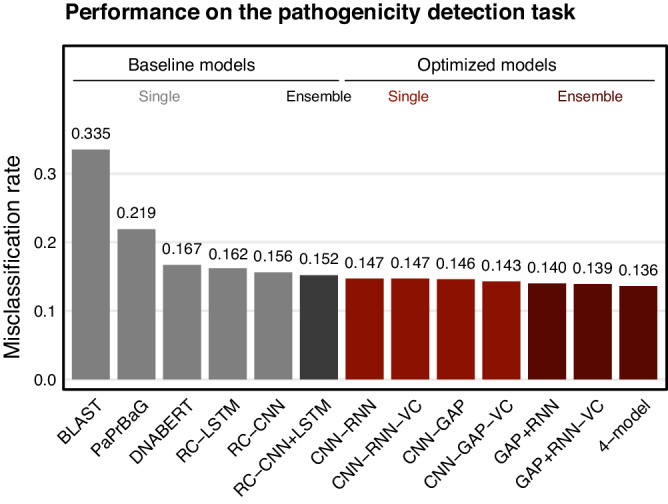# Author Correction: Optimized model architectures for deep learning on genomic data

**DOI:** 10.1038/s42003-024-06318-y

**Published:** 2024-05-23

**Authors:** Hüseyin Anil Gündüz, René Mreches, Julia Moosbauer, Gary Robertson, Xiao-Yin To, Eric A. Franzosa, Curtis Huttenhower, Mina Rezaei, Alice C. McHardy, Bernd Bischl, Philipp C. Münch, Martin Binder

**Affiliations:** 1 Department of Statistics, LMU Munich, Munich, Germany; 2 Munich Center for Machine Learning, Munich, Germany; 3 Department for Computational Biology of Infection Research, Helmholtz Center for Infection Research, 38124 Braunschweig, Germany; 4 Braunschweig Integrated Centre of Systems Biology (BRICS), Technische Universität Braunschweig, Braunschweig, Germany; 5 Department of Biostatistics, Harvard School of Public Health, Boston, MA USA; 6 German Centre for Infection Research (DZIF), partner site Hannover Braunschweig, Braunschweig, Germany

**Keywords:** Machine learning, Classification and taxonomy, Computational models, Bioinformatics

Correction to: *Communications Biology* 10.1038/s42003-024-06161-1, published online 30 April 2024

The original version of the Article contained an error in the annotations of the bars in Fig. 4.

This has now been corrected in the PDF and HTML versions of the Article.

Original figure:
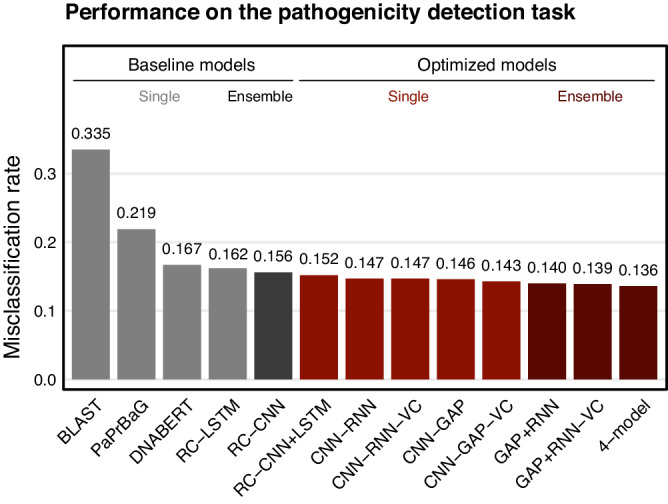


Corrected figure: