# Supplementary material for: Optimized model architectures for deep learning on genomic data
Source: Commun Biol. 2024 Apr 30;7:516. doi: 10.1038/s42003-024-06161-1 (PMC11063068; doi:10.1038/s42003-024-06161-1)
Supplement: Supplementary file 2 — Supplementary Information [file 42003_2024_6161_MOESM2_ESM.pdf]

Supplementary Material

Supplementary Results

Task	Viral Classification								Pathogenicity Detection	
Sequence Length	150				10,000				250	
Metric	CNN-GAP-2h	**CNN-GAP-6h**	CNN-RNN-2h	CNN-RNN-6h	CNN-GAP-2h	CNN-GAP-6h	**CNN-RNN-2h**	CNN-RNN-6h	**CNN-GAP**	CNN-RNN
Learning Rate	0.0002295	**0.0002138**	0.0002630	0.0014382	0.0003255	0.00027436	**0.0001168**	0.0004349	**0.0002418**	0.0003251
RC as Additional Input	True	**True**	True	True	False	True	**True**	False	**True**	True
Optimizer	Adam	**Adam**	Adam	Adam	Adam	Adam	**Adam**	Adam	**Adam**	Adam
Model Type	GAP	**GAP**	RNN	RNN	GAP	GAP	**RNN**	RNN	**GAP**	RNN
Number of Conv. Layers (n_c)	6	**9**	5	3	5	5	**4**	1	**6**	5
Number of Conv. Blocks (n_{cb})	7	**7**	3	4	5	7	**9**	10	**7**	3
First Kernel Size (k_0)	3.7992	**4.1379**	2.6317	6.0517	7.8576	15.0923	**2.8262**	12.8539	**4.1487**	2.6463
Last Kernel Size (k_{end})	20.9910	**44.4341**	5.4452	53.4114	2.5291	2.6436	**2.7787**	58.9992	**19.2934**	3.4839
First Number of Filters (f_0)	777.8104	**622.1899**	262.3433	378.8905	146.1158	108.9821	**60.1536**	708.3316	**697.8605**	147.8401
Last Number of Filters (f_{end})	22.3111	**29.8014**	901.9673	57.5173	370.7959	117.3769	**401.0379**	147.4949	**49.6290**	748.9535
Last Dilation Factor (d_{end})	2.1027	**3.3495**	1.9187	3.2531	11.1640	22.2875	**6.0812**	57.2750	**2.7963**	4.6694
Total Max-Pooling (p_{end})	15.4875	**15.7883**	14.7550	7.0775	69.7147	83.9126	**115.4169**	90.8419	**21.0560**	3.1384
Momentum of Batch-Norm	0.6888	**0.7605**	0.4730	0.1840	0.7577	0.8495	**0.1288**	0.1993	**0.6945**	0.0261
Leaky-ReLU Alpha Value	0.2630	**0.0481**	0.3142	0.0302	0.8579	0.8018	**0.8324**	0.2514	**0.3399**	0.5381
Residual Block	False	**False**	False	True	False	False	**True**	True	**False**	False

Number of Dense Layers	1	**2**	0	0	2	2	**1**	0	**1**	1
Units of Dense Layers	79	**284**	1474	32	365	470	**152**	73	**101**	1033
Dropout	0.1844	**0.2257**	0.3145	0.2108	0.1406	0.1424	**0.3110**	0.6830	**0.1773**	0.3122
Activation of Dense Layers	tanh	**tanh**	tanh	ReLU	ReLU	ReLU	**tanh**	tanh	**tanh**	tanh
Recurrent Type	-	**-**	LSTM	LSTM	-	-	**LSTM**	LSTM	**-**	LSTM
Number of Rec.Layers	-	**-**	3	1	-	-	**1**	1	**-**	1
Uni-/Bi- dir, Rec. Layers	-	**-**	True	True	-	-	**True**	True	**-**	True
Recurrent Units	-	**-**	19	58	-	-	**452**	23	**-**	65
Skip Ratio for GAP (r_s)	0.6496	**0.7169**	-	-	0.6063	0.5176	**-**	-	**0.6457**	-
Class-Balanced Accuracy	0.7609	**0.7978**	0.7788	0.7905	0.9870	0.9864	**0.9879**	0.9859	**0.8541**	0.8525
Number of Parameters	2,675,616	**3,471,893**	8,050,761	1,606,250	1,367,073	714,802	**3,704,628**	175,426	**3,226,246**	3,757,059

Supplementary Table 1: Specific hyperparameter values and performance of chosen network architectures. Shown are the optimized hyperparameters for both CNN-GAP and CNN-RNN models and both viral classification and pathogenicity detection tasks. For the viral classification task, models are optimized and evaluated to discriminate between bacteria, eukaryotic viruses, and prokaryotic viruses with two-hour and six-hour runs. 2h and 6h represent the training time allocated to each run during the hyperparameter optimization, where the 6h optimization was warm-started using the results of the 2h optimization. For the pathogenicity detection task, only two-hour runs are optimized. The evaluation was performed separately for sequence lengths of 150 nt (viral detection), 10,000 nt (viral detection), and for the pathogenicity classification task. The class-balanced accuracy was evaluated on a test set not seen during optimization. The best architectures in terms of class-balanced accuracy are shown in bold for all sequence length values.

Model/Baseline	Balanced Accuracy	Length (nt)
Bowtie2[1]	71.7%	150
Kraken2[2]	76.0%	
Best ML baseline (Fiannaca[3])	75.0%	
Optimized model	**79.8%**	
Minimap2	68.1%	10k
Kraken2[2]	89.4%	

Best ML baseline (Fiannaca[3])	98.6%	
Optimized model	**98.8%**	

Supplementary Table 2: Balanced accuracy of baselines for the viral classification task, including non-machine learning baselines. The optimized GenomeNet-Architect model outperforms all baseline models. The bold values represent the best models.

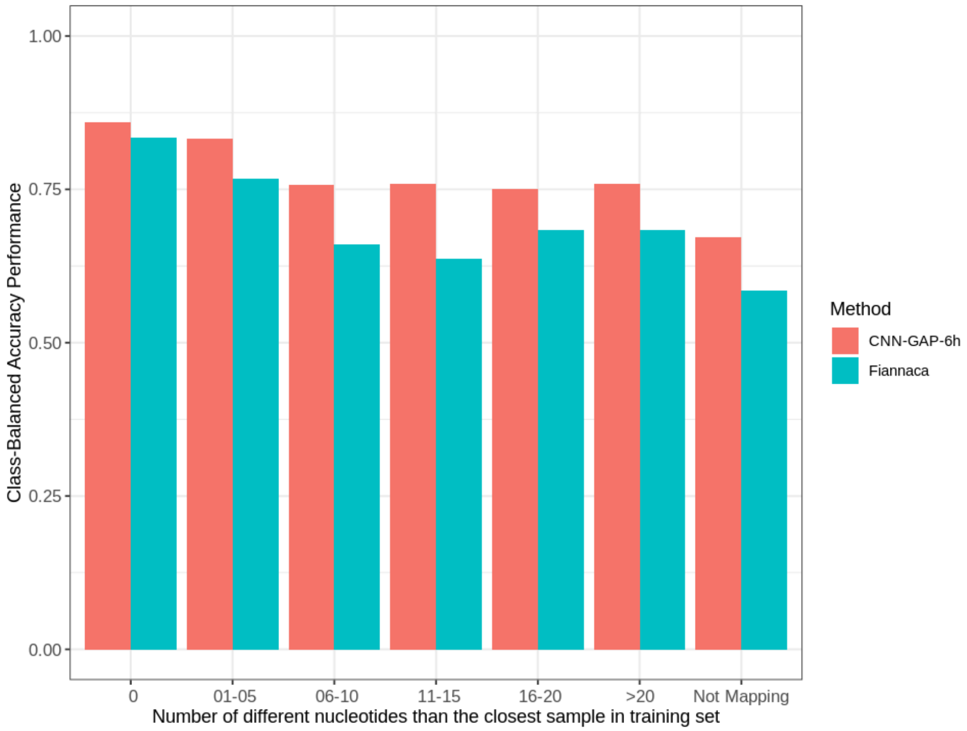

Supplementary Figure 1: Model performance stratified similarity groups based on the closest sample in the training set via Bowtie2[1] alignment. We binned the samples based into the following groups: exact match (alignment) of a subsample from the test set to the combined training dataset (all groups); 1-5, 6-10, 11-15, and 16-20, and differences of more than 21 nucleotides (nt), respectively. The final bin denotes samples from the test set that had no alignment at all to the training database, denoted as not aligned (NA) group. We processed these sets from the test datasets using methods to the best-performing baseline Fiannaca and the optimized method CNN-GAP-6h. While by definition, all samples in the NA group fail to be processed by the alignment-based baseline, our optimized model CNN-GAP-6h outperformed the best-performing baseline model Fiannaca[3] by 8.6 percentage points. Furthermore, our optimized model showed a better performance compared to Fiannaca[3] in all bins. When tested simulated reads that have an Illumina error profile, our optimized models outperform Fiannaca by 10.5 percentage points.

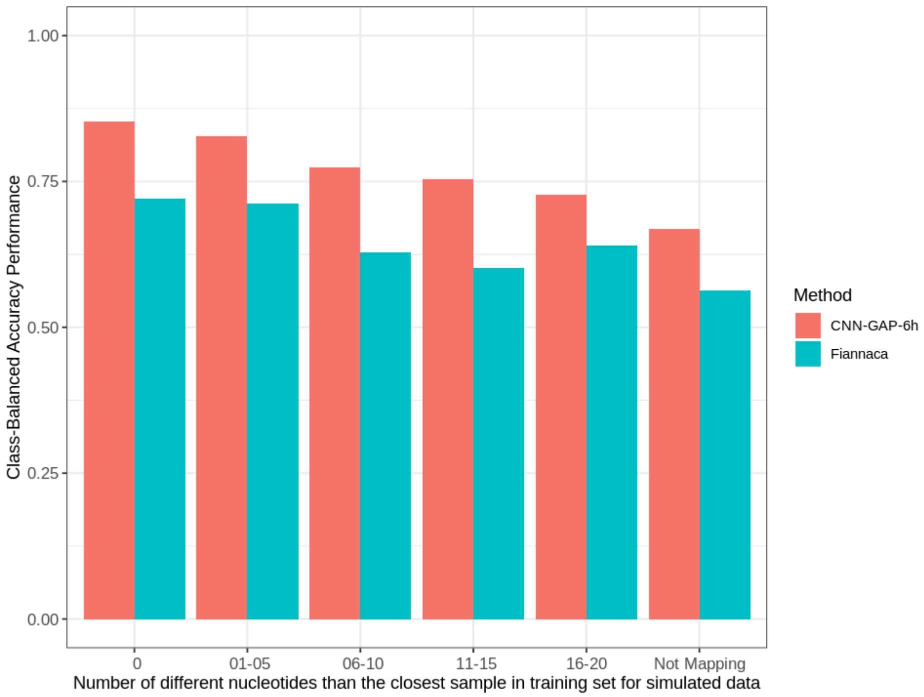

Supplementary Figure 2: Model performance stratified similarity groups based on the closest sample in the training set via Bowtie2[1] alignment for simulated data. To ensure the robustness and applicability of our model to real sequencing scenarios, we conducted experiments with synthetic reads that included simulated error profiles. These synthetic reads were generated using the NGSNGS[4] tool, a tool designed to simulate sequencing reads with realistic error patterns. This approach allowed us to model the types of inaccuracies typically encountered in high-throughput sequencing technologies, such as substitutions, insertions, and deletions, thereby providing a more challenging and realistic test environment for our model.

Supplementary Note 1: Longer Optimization Runs. The optimization procedure can be restarted several times with increasing values of t (training time of each run in hyperparameter optimization), using all previous points as warm start data. After setting the training time of the models to 2 hours and then to 6 hours in the hyperparameter optimization, we further increased the training time to 20 hours. This optimization evaluated 528 more configurations, added 33.1 days of training, and thus increased the total hyperparameter optimization duration by 170%. However, we did not observe any increase in the accuracy performance for the sequence length of 150. For the sequence length of 10,000, the class-balanced misclassification error rate decreased from 1.21% to 1.11%, while the number of parameters also decreased from 3.7 million to 0.8 million (CNN-RNN-2h vs CNN-RNN-20h). However, we do not find this increase compelling, as a 0.1 percentage point change is rather small, considering the substantial increase in total optimization run-time.

Supplementary References

1. Langmead, B. & Salzberg, S. L. Fast gapped-read alignment with Bowtie 2. *Nat. Methods* **9**,

357–359 (2012).

2. Wood, D. E. & Salzberg, S. L. Kraken: ultrafast metagenomic sequence classification using exact alignments. *Genome Biol.* **15**, 1–12 (2014).
3. Fiannaca, A. *et al.* Deep learning models for bacteria taxonomic classification of metagenomic data. *BMC Bioinformatics* **19**, 198 (2018).
4. Henriksen, R. A., Zhao, L. & Korneliussen, T. S. NGSNGS: next-generation simulator for next-generation sequencing data. *Bioinformatics* **39**, (2023).
