# Supplementary material for: Optimized model architectures for deep learning on genomic data
Source: Commun Biol. 2024 Apr 30;7:516. doi: 10.1038/s42003-024-06161-1 (PMC11063068; doi:10.1038/s42003-024-06161-1)
Supplement: Supplementary file 3 — Description of Additional Supplementary Files [file 42003_2024_6161_MOESM3_ESM.pdf]

Description of Additional Supplementary Files

File name: Supplementary Data 1

Description: Source data for Figure 3 and Supplementary Figures 1 and 2.
